# Body Mass Index (BMI) and Its Influence on the Cardiovascular and Operative Risk Profile in Coronary Artery Bypass Grafting Patients: Impact of Inflammation and Leptin

**DOI:** 10.1155/2020/5724024

**Published:** 2020-06-23

**Authors:** Katja Buschmann, Julius Wrobel, Ryan Chaban, Romina Rösch, Ahmed Ghazy, Alina Hanf, Katrin Schäfer, Andreas Daiber, Andres Beiras-Fernandez, Christian Friedrich Vahl

**Affiliations:** ^1^Department of Cardiothoracic and Vascular Surgery, University Medical Center of the Johannes Gutenberg-University Mainz, 55131 Mainz, Germany; ^2^Center for Cardiology, Cardiology I, University Medical Center of the Johannes Gutenberg-University Mainz, 55131 Mainz, Germany; ^3^German Center for Cardiovascular Research (DZHK), partner site Rhine-Main, Mainz, Germany

## Abstract

**Background:**

Obesity is related to coronary artery disease (CAD) and worse outcomes in coronary artery bypass graft (CABG) patients. Adipose tissue itself is an endocrine organ that secretes many humoral mediators, such as adipokines, which can induce or reduce inflammation and oxidative stress.

**Objectives:**

We investigate the relationship between the body mass index (BMI), inflammation, and oxidative stress by measuring serum levels of leptin, interleukin-6, and 3-nitrotyrosine in CABG patients and correlate their levels to the cardiovascular and operative risk profiles.

**Methods and Results:**

45 men (<75 years) with a median BMI of 29 (21-51) kg/m^2^, who were diagnosed with CAD and scheduled for elective CABG, were included after applying the following exclusion criteria: prior myocardial infarction, reoperation, female gender, and smoking. Patients' blood samples were taken preoperatively. Several markers were measured. We found significant correlations between leptin and BMI (*p* < 0.0001) as well as between leptin and 3-nitrotyrosine (*p* = 0.006). Interleukin-6 was correlated with C-reactive protein (*p* < 0.0001) and with the incidence of insulin-dependent diabetes mellitus (*p* = 0.036), arterial hypertension (*p* = 0.044), reduced left ventricular function (*p* = 0.003), and severe coronary calcification (*p* = 0.015). It was also associated with significantly longer extracorporeal bypass time (*p* = 0.009). Postoperative deep sternal wound infections could be predicted by a higher BMI (*p* = 0.003) and leptin level (*p* = 0.001).

**Conclusions:**

There seems to be a correlation between inflammatory processes and cardiovascular morbidity in our cohort. Further, the incidence of deep sternal wound infections is related to a higher BMI and leptin serum level.

## 1. Introduction

Adiposity is well-known as a cardiovascular risk factor. Obese coronary artery bypass graft (CABG) patients are associated with higher postoperative morbidity and worse long-term survival [1]. Nevertheless, the body mass index (BMI) itself does not play a role in preoperative risk adjustment scores for cardiac surgery so far [2]. In a former experimental study, we could prove a correlation of BMI with a negative inotropic effect in an isolated human myocardium [3]. The adipose tissue itself is the largest endocrine organ of the human body and secrets more than 150 adipokines with mostly proinflammatory and cardiovascular potent mediation [4–8].

Clinical studies in humans showed that obesity—defined by raised BMI—is associated with elevated serum levels of the adipokine leptin [9]. Coronary artery disease (CAD) is also associated with increased serum leptin levels [10–12]. Leptin levels are identified as a malignant predictor of mortality and morbidity of patients with CAD [13]. Wallace et al. showed that women have significantly higher leptin levels than men [11]. Therefore, we decided to determine the serum leptin levels only in male CABG patients to avoid gender bias and to achieve more homogeneity in the measured leptin level. In addition, leptin has a direct proinflammatory effect on T cells and monocytes, leading to the release of cytokines such as tumor necrosis factor-*α* (TNF-*α*) and interleukin 6 (IL-6) [14–16]. Furthermore, leptin is associated with the inflammation marker C-reactive protein (CRP) [15], and high levels of these biomarkers (leptin, IL-6, and CRP) are predictive for worse outcome in patients with CAD [15]. Leptin also induces the formation and the intracellular accumulation of reactive oxygen species (ROS) [15]. In order to evaluate the referred association of inflammation and leptin, we planned to measure not only the standard serum CRP but also the IL-6 levels.

The aim of this study is to establish a correlation between the humoral expression of leptin and the inflammatory and oxidative status of obese patients undergoing CABG, as well as the clinical influence of these biomarkers.

## 2. Materials and Methods

### 2.1. Patients

**Figure 1:**
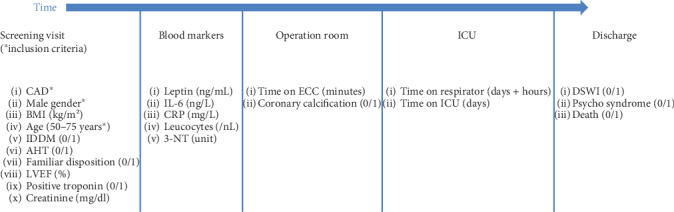
Flow chart of the time point of determination and documentation for each parameter/data ([0/1] = [No/yes]; 3-NT = 3-nitrotyrosine (rel. IOD, % of control normalized to loading control); AHT = arterial hypertension; BMI = body mass index; CRP=C-reactive protein; d = days; DSWI = deep sternal wound infection; ECC = extracorporeal circulation; h = hours; ICU = intensive care unit; IDDM = insulin-dependent diabetes mellitus II; IL-6 = interleukin 6; LVEF = left ventricular ejection fraction).

This study was approved by the Institutional Review Board (processing number 837.051.15 (9819), 23 March 2015). A total of 45 consecutive elective isolated CABG patients with CAD and a median BMI of 29 [21-51] kg/m^2^, male gender, and an age between 50 and 75 years (67 ± 8.4 years) were included. Exclusion criteria were prior myocardial infarction, reoperation, female gender, and smoking.  Figure 1 shows the timing of the screening visit, the evaluation and documentation of preoperative characteristics, preoperative blood markers, and the final documentation of intraoperative and intensive care unit (ICU) data as well as the discharge data for the final recording of deep sternal wound infection (DSWI), as a postoperative complication. The observation period ended with the discharge of the patient. We interviewed the study patients for their age and the presence of cardiovascular risk factors as insulin-dependent diabetes mellitus (IDDM), familiar disposition, and arterial hypertension (AHT). Further comorbidities such as renal dysfunction, assessed by the creatinine serum level, and left ventricular ejection fraction (LVEF) were recorded. Blood samples were obtained simultaneously with the regular clinical blood sampling at administration, usually 24 hours before surgery. We measured leptin, IL-6, CRP, leukocytes, and 3-nitrotyrosine (3-NT). The duration of extracorporeal circulation (ECC) is relevant for postoperative outcome and therefore reported as well as the diagnosis of a severe coronary calcification, assessed by the cardiac surgeon in his operation report. The time on respirator and stay length on ICU were documented. Clinical assessments and documentations were used for the query of DSWI, a therapy relevant psychosyndrome and death.

### 2.2. Blood Biomarkers

Patients' blood samples were taken preoperatively each with 3 serum pipes. IL-6, CRP, and leukocytes were measured by the intern laboratory medicine. The other samples were frozen and collected for leptin enzyme-linked immunosorbent assay (ELISA) and oxidative stress level determination. Serum leptin levels were determined using a commercially available Human Leptin Quantikine ELISA Kit (R&D Systems), following the instructions of the manufacturer.

### 2.3. 3-Nitrotyrosine Levels (3-NT)

Levels of 3-NT and 8-isoprostane, as markers of oxidative stress, were determined in human serum using a commercial ELISA kit (human immunoassay, 3-NT: #CSB-E14324h, Cusabio, Wuhan, Hubei Province 430206, China; 8-isoprostane: #MBS109360, San Diego, CA, USA) following the instructions of the vendor and previously [17]. Protein tyrosine nitration was also determined by dot blot analysis using a specific antibody for 3-NT (Millipore 05-233 Mouse mAb, 1 : 1000 in PBS/5% Milk) [17, 18]. Briefly, 100 *μ*l (0.5 *μ*g/*μ*l protein based on Bradford analysis) of the ethylenediaminetetraacetic acid (EDTA) plasma was transferred to a Protran BA85 (0.45 *μ*m) nitrocellulose membrane (Schleicher & Schuell, Dassel, Germany) using a Minifold I vacuum Dot-Blot system (Schleicher & Schuell, Dassel, Germany). Each slot was washed twice with 200 *μ*l phosphate-buffered saline (PBS) before and after protein transfer. The membrane was dried for 60 min at 60°C. Positive bands were detected by enhanced chemiluminescence after incubation with peroxidase-coupled anti-mouse or rabbit secondary antibodies for 3-NT-positive proteins or transferrin, respectively (goat-anti-mouse-peroxidase-labeled and goat-anti-rabbit-peroxidase-labeled, 1 : 10,000) (Vector Laboratories, CA, USA). All incubation and washing steps were performed according to the manufacturer's instructions. Densitometric quantification of the dots was performed using the SuperSignal-enhanced chemiluminescence kit from Thermo Scientific. The densitometric 3-NT signals were normalized to either Ponceau S staining or to the densitometric signals of the serum loading control transferrin (anti-transferrin antibody (Thermo Fisher PA3-913); rabbit pAb, 1 : 5,000 in PBS-T/5% BSA) staining (rel. IOD, % of control).

### 2.4. Statistical Analysis

Statistical description and analyses were performed using IBM SPSS 23 (IBM, USA). Quantitative variables were expressed as the mean ± standard deviation if they were normally distributed (skewness between -1 and 1) and as median with range if they were not normally distributed (skewness < −1 and>1). For nonparametric data, the Mann–Whitney *U* or the exact Fisher test were used; for parametric data Student's *t*-test. Linear regression was used for the assessment of correlation between metric parameters. An alpha-value of 0.05 was chosen for significance. The cut-off values were determined within an ROC analysis. As cut-off, the values with the smallest Euclidian distance to the upper left of the plot were taken.

## 3. Results

### 3.1. Blood Markers and Hospitalization Data

BMI and all measured blood markers showed a wide variation: BMI 29 (21-51) kg/m^2^, leptin 9.157 (1.594-87.984) ng/mL, IL-6 16.5 (2-79) ng/L, CRP 5.3 (0.29-291) mg/L, leucocytes 8.2 (4.46-24.3) cells/nL, and 3-NT 53.24 (21.26-187.20) (rel. IOD, % of control). Time on ECC was 71.14 ± 20.77 minutes. Severe coronary calcifications were assessed in 21 CABG operations. The postoperative time on respirator was 10 (1-420) hours and stay on ICU was 2.54 ± 3.69 days. With the discharge of the patient to home or to rehabilitation 5 cases of DSWI, no psycho syndrome and no death were recorded.

### 3.2. Inflammation Markers and Preoperative Risk Profile

**Figure 2:**
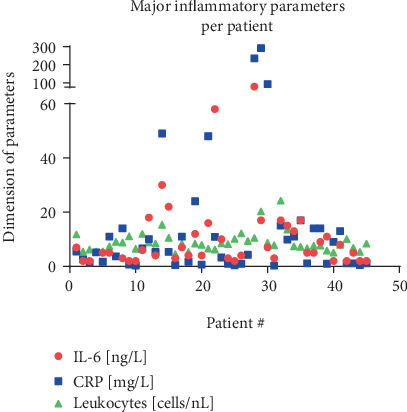
All 45 individual values for the measured parameters of inflammation are illustrated.

**Figure 3:**
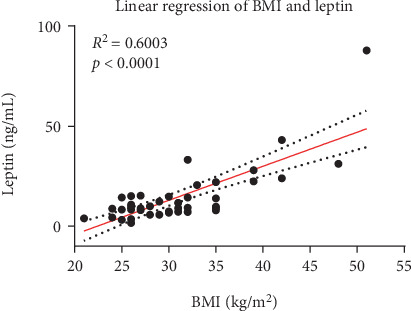
Significant correlation of BMI with leptin in 45 patients.

**Figure 4:**
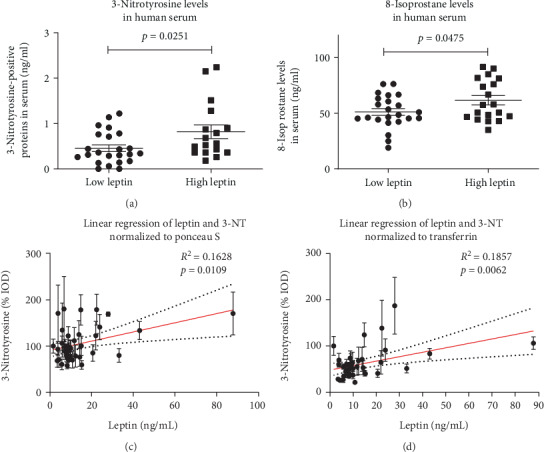
Determination of the oxidative stress parameters 3-nitrotyrosine (3-NT) (a) and 8-Isoprostane (b) by commercial ELISA assays of a proof of increased reactive oxygen and nitrogen species formation in patients with leptin levels higher than the median. Data are mean ± SEM. (c, d) Significant correlation of leptin and levels of the serum oxidative stress marker 3-NT in 40 patients (*p* values and correlation coefficients are provided in the graphs). Measured 3-NT staining was either normalized to Ponceau S staining (c) or transferrin content (d).

**Figure 5:**
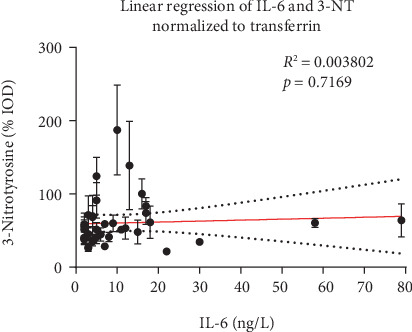
Correlation of IL-6 and levels of the serum oxidative stress marker 3-nitrotyrosine (3-NT) in 40 patients (correlation coefficient is provided in the graph).

Parameters of inflammation showed a significant correlation as CRP was significantly correlated with IL-6 (*p* < 0.0001, *R*^2^ = 0.3) and leukocytes (*p* = 0.003, *R*^2^ = 0.186) (Figure 2). On the other hand, IL-6 serum levels were not affected by the BMI (*p* = 0.821, *R*^2^ = 0.001) and did not correlate with serum leptin levels (*p* = 0.608, *R*^2^ = 0.007). Leptin was significantly correlated with BMI (*p* < 0.0001, *R*^2^ = 0.6) (Figure 3) and also with the oxidative stress marker 3-NT (*p* = 0.0109, *R*^2^ = 0.1628 or *p* = 0.0062, *R*^2^ = 0.1857, depending on the normalization method) (Figure 4). In a pilot experiment, we confirmed that patients with high leptin levels have higher oxidative stress parameters (3-NT and 8-isoprostane). The distribution pattern also varied to some extent in dependence of the normalization method, but the overall trend was reproducible in both measurements. 3-NT was not significantly correlated with IL-6 (*p* = 0.7169, *R*^2^ = 0.0038) (Figure 5).

**Figure 6:**
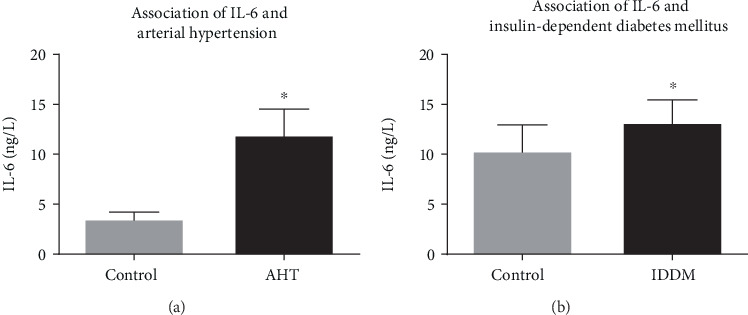
IL-6 levels in patients with (a) AHT (*n* = 39) and (b) IDDM (*n* = 7) compared to IL-6 levels in patients without arterial hypertension (no AHT/control (a), *n* = 6) and without IDDM (no IDDM/control (b), *n* = 38). IL-6 levels were significantly higher in patients with AHT (3.5 ± 0.7 vs 11.9 ± 2.6 ng/l) and with IDDM (10.3 ± 2.6 vs 13.2 ± 2.3 ng/l). Data are mean ± SEM.

Preoperative left ventricular ejection fraction was inversely correlated with IL-6 (*p* = 0.003, *R*^2^ = 0.202), CRP (*p* = 0.005, *R*^2^ = 0.171), and leucocytes (*p* = 0.005, *R*^2^ = 0.170), whereas patients with IDDM showed significantly higher level of IL-6 (*p* = 0.036) as well as patients with AHT (*p* = 0.044) (Figure 6). Furthermore, preoperative creatinine was proportional correlating with leptin (*p* = 0.001, *R*^2^ = 0.223) and also significantly higher in patients with positive troponin (*p* = 0.003). Patients' age showed no statistical relation to IDDM, AHT, or familiar disposition.

### 3.3. Inflammation Markers and Coronary Sclerosis

**Figure 7:**
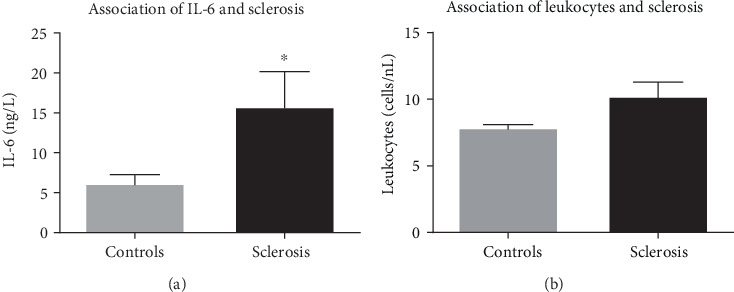
IL-6 levels (a) and leucocytes (b) compared in patients with intraoperative status of severe coronary calcification (sclerosis, black) or normal coronary sclerosis (control, grey). Patients with severe coronary calcification had significantly higher levels of IL-6 (6.1 ± 1.1 vs 15.8 ± 4.4 ng/l), whereas leucocytes were only increased by trend (7.7 ± 0.4 vs 10.2 ± 1.1 cells/ng, *p* = 0.085). Data are mean ± SEM, *n* = 21 − 24 patients.

Severe coronary sclerosis was assessed in 21 patients; these patients did not show a significantly higher blood level of leucocytes, but were outstanding with significantly higher blood levels of IL-6 (*p* = 0.015) (Figure 7). Patients' age, creatinine value, and LVEF were neither significant for the CVR profile nor for the severity of coronary calcification (Table 1). Patients with reduced LVEF did show the trend of a higher rate of severe coronary calcification (*p* = 0.09, Table 1) In addition to this, patients with severe coronary sclerosis had a significantly higher rate of IDDM (*p* = 0.031), and bypass grafting operation demanded significantly longer time on ECC (63.57 minutes versus 79.85 minutes, *p* = 0.009). The time on respirator was correlated with BMI (*p* = 0.042, *R*^2^ = 0.104) and CRP (*p* = 0.024, *R*^2^ = 0.127) and significantly longer in patients with IDDM (*p* = 0.004). Furthermore, patients with IDDM did show a prolonged stay on ICU (*p* = 0.008).

**Figure 8:**
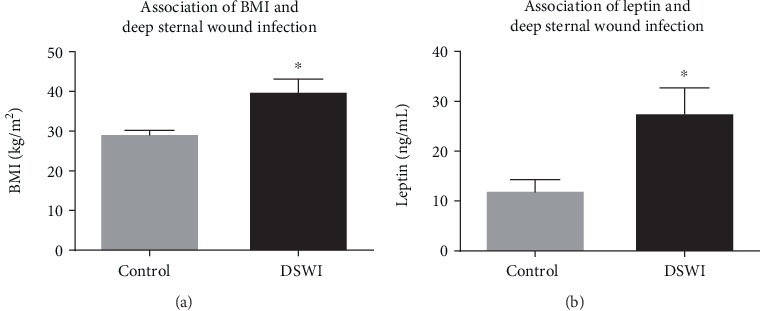
BMI (a) and leptin levels (b) in patients with deep sternal wound infection (black, DSWI, *n* = 5) versus the control (grey) group (*n* = 40). Patients with DSWI had significantly higher BMI (29.4 ± 0.9 vs 40.0 ± 3.1 kg/m^2^) and significantly raised levels of leptin (12.2 ± 2.2 vs 27.7 ± 5 ng/l). Data are mean ± SEM.

There were 5 cases of DSWI. Reliable DSWI markers were leptin (*p* = 0.001) and BMI (*p* = 0.003) (Figure 8). Patients with DSWI were significantly more often diabetic patients (*p* = 0.021) but did neither exceed in time on respirator nor on intensive care unit.

### 3.4. Analysis of Cut-Off Values

The inflammatory marker IL-6 delivers cut-off values for the cardiovascular and operative risk profile by significant correlation with IDDM, AHT, and severe coronary calcification whereas leptin and BMI are predictive for DSWI (Table 2).

## 4. Discussion

### 4.1. Main Findings and Clinical Relevance

This study investigated the association of the inflammatory marker IL-6 with the cardiovascular and operative risk profile of CABG patients. We found that IL-6 is associated with the cardiovascular (IDDM and AHT) as well as for the operative risk profile (inversely correlated with LVEF and severe coronary calcification). This finding is in accordance with the reported low-grade inflammatory phenotype of IDDM and AHT [19]. The clinical importance of IDDM and AHT is further supported by large epidemiological studies ranking them among the leading cardiovascular risk factors and causes of premature death at the global level [20]. AHT and its correlation with inflammation markers (CRP or IL-6) have been also described in literature [21]. Patients with reduced LVEF show the trend to have more often a severe coronary sclerosis. In the present study, the inflammation markers IL-6 and CRP and the number of leucocytes were significantly correlated. Moreover, we demonstrate a correlation between leptin and BMI and that both parameters are associated with DSWI as an operative complication risk.

### 4.2. CABG Surgery and Inflammation

Severe coronary calcification (reported by the surgeon itself) is accompanied by significantly longer time on ECC and thereby increasing operative risk. Also the leucocytes were significantly correlated with the status of severe coronary calcification in the present study, similar to the inflammation marker IL-6. Although, previous data do not support a significant correlation of coronary calcification and CRP levels in CABG patients, a subgroup analysis in statin-free patients found a clear correlation [22]. Another recent study has proven the association between fibrinogen level, an acute phase protein, and a parameter of inflammation, and the severity of coronary stenosis [23]. CRP is significantly correlated with the time on respirator [24]. Thus, elective CABG patients with preoperatively raised CRP values should firstly be searched for an infective pulmonary focus and optionally be treated with antibiotic therapy until surgery can be planned. Although higher preoperative CRP levels are mostly related to infections [25], also other conditions such as schizophrenia, cardiogenic shock, and autoimmune conditions, including rheumatoid arthritis, lupus, and certain types of inflammatory bowel disease, such as Crohn's disease and ulcerative colitis, may be associated with increased CRP levels.

### 4.3. CABG Surgery and Leptin

Our determination of leptin in this cohort of patients is completely coherent with the literature. Considine et al. describe leptin levels in obese with 31.3 ± 24.1 ng/mL and about 7.5 ± 9.3 ng/mL in normal-weighted humans [9], and Wolk et al. reported leptin levels with median 9.2 ng/mL [0.7-90.6 ng/mL] in patients with established CAD [12]. Both are similar to our results with median 9.157 (1.594-87.984) ng/mL. Furthermore, Wolk et al. reported that the leptin level is independent of other risk factors, e.g., lipid status and CRP. In this clinical study, the systemic leptin level was also not correlated with markers of inflammation. This can be caused by many other relevant effects of pro- and anti-inflammatory adipokines. Furthermore, we observed increased oxidative stress in patients with systemic higher levels of leptin. This can be explained by the fact that oxidative stress reduces the production of beneficial, anti-inflammatory adipokines [26]. In line with this notion, oxidative stress negatively affects the collagen turnover and supports the synthesis of collagen, for example, in the heart, resulting in worse LVEF by the remodeling process [27]. In a former experimental study, we found also signs of fibrotic remodeling in septic/inflammatory cardiomyopathy [28]. A more recent study proposed the leptin/adiponectin ratio as another parameter of prognostic importance besides markers of inflammation [29].

### 4.4. CABG Surgery and BMI

In our study, leptin is significantly correlated with the BMI. However, there are clear difficulties reported for the leptin level dependence on BMI [30]. In addition, the development of leptin resistance due to insulin resistance in diabetic and adipose patients may contribute to a rather reduced leptin effect [30, 31]. Regarding the here established significant correlation of BMI and leptin, it should be stressed that the preoperative BMI in CABG patients was associated with DSWI, a major complication in CABG surgery, and thereby may confer some prognostic value to BMI as well as leptin levels (BMI > 37 kg/m^2^ cut-off, Table 2). This is further supported by the significant correlation of leptin with the preoperative creatinine levels in our patient collective. This relation is also described by others [21, 31]. Also, previous studies reported on a higher risk for major complications among CABG patients with increasing BMI [32, 33].

### 4.5. Leptin, Inflammation, Arterial Hypertension, and Diabetes Mellitus

Tsai et al. showed a correlation between AHT and leptin predicting arterial stiffness in hypertensive patients [21]. In our results, we could not show any correlation between leptin and AHT. One explanation could be the bias of IDDM—at least seven patients in our study collective showing decreased leptin levels [30, 31]. Nevertheless, both of our study groups—with or without IDDM—show median leptin levels with 9.2/9.1 ng/mL. There is no effect of age (Table 1) upon the severity of coronary calcification. But patients with a preoperatively reduced LVEF showed the trend of a higher rate of severe coronary calcification—accompanied with and presumably caused by raised inflammation (marker IL-6). Nevertheless, inflammation markers in the blood seem to have more influence resulting in cardiovascular damage like AHT and coronary calcification.

The significance of the preoperative creatinine level was neither demonstrated in our study nor in another recent longitudinal study [34]. In our work, there was a significantly higher rate of severe coronary calcification in patients with IDDM. A recent study with the optical coronary tomography method [35] could exclude any impact of type 2 diabetes mellitus on coronary calcification but has proven the burden of a plaque rupture in type 2 diabetes mellitus. The definition of type 2 diabetes mellitus was Hba1c > 6.5% or intake of oral antidiabetic drugs. In our study. the criteria were stronger with the insulin-dependency. Thus, both different results are not comparable.

One explanation could be that there are pro- and anti-inflammatory adipokines that, depending on their expression profile, promote or suppress inflammation. In this study, we did only measure the proinflammatory leptin without measurement of an antagonist, e.g., the anti-inflammatory adiponectin. Instead, we concentrated on the consequences of inflammation by measurement of the oxidative stress marker 3-NT and its correlation with IL-6. To our surprise, we could not show a positive correlation between IL-6 and oxidative stress (Figure 5).

### 4.6. Limitations of the Study

A major limitation of our study is the small cohort size, thus limiting the power of our findings and resulting in small effect sizes. Previous studies were based on several hundreds of individuals, e.g., the investigation of risk factors for surgical wound infections and postoperative bacteremia for patients undergoing CABG surgery (*n* = 693) [36]. In addition, our observation period was quite short and ends with the discharge from hospital. Similar studies used longer follow-up periods of up to five years, e.g., investigation of impact of BMI and the associated biomarkers on clinical outcomes after CABG (*n* = 234) [32].

## 5. Conclusion

In our study, neither the BMI and nor the leptin level are as relevant as the systemic inflammation markers that are probably responsible for the worse prognosis and high cardiovascular event rate in obese CABG patients. Our observations are in good accordance with meta-analysis reporting pre- and perioperative inflammation to be major determinants of prognosis among CABG patients [37]. This may also explain why modern antidiabetic drugs with clearly characterized anti-inflammatory properties show beneficial effects on prognosis among CABG patients. Examples are the therapy with a sodium glucose cotransporter 2 inhibitor [38], glucagon-like peptide-1 [39], statins [40, 41], or even a specific anti-inflammatory, monoclonal-based drug [42].

## Figures and Tables

**Table 1:** Patients' comorbidities (age, creatinine, and left ventricular function) are not significantly influencing the cardiovascular risk profile (IDDM, familiar disposition, and arterial hypertension) or significantly correlating with the intraoperative finding of a severe coronary calcification. However, there is a significant relation between the value of the left ventricular function (LVEF %) and severe coronary calcification.

	Insulin-dependent DM [0: *n* = 38, 1: *n* = 7]	Familiar disposition [0: *n* = 33, 1: *n* = 12]	Arterial hypertension [0: *n* = 6, 1: *n* = 39]	Severe coronary calcification [0: *n* = 24, 1: *n* = 21]
Age (years)	*p* = 0.58	*p* = 0.97	*p* = 0.66	*p* = 0.89
Creatinine (mg/dL)	*p* = 0.95	*p* = 0.60	*p* = 0.79	*p* = 0.82
LVEF (%)	*p* = 0.87	*p* = 0.38	*p* = 0.71	*p* = 0.09

**Table 2:** Statistically tested cut-off values of markers of inflammation (IL-6) and adiposity (body mass index and leptin) for the presence of insulin-dependent diabetes as a cardiovascular risk factor, the intraoperatively finding of a severe coronary calcification as an operative risk profile, and the happening of a postoperatively deep sternal wound infection.

Cut-off values	Insulin-dependent DM	Arterial hypertension	Severe coronary calcification	Deep sternal wound infection
IL-6 (ng/L)	15.5	6.5	12.5	
BMI (kg/m^2^)				37.0
Leptin (ng/mL)				23.17

## Data Availability

The data used to support the findings of this study are available from the corresponding author upon request.

## Abbreviations

[0/1]: No/yes
AHT: Arterial hypertension
BMI: Body mass index (body weight (kilogram)/body height (m)^2^)
CABG: Coronary artery bypass graft
CAD: Coronary artery disease
CRP: C-reactive protein
CVR: Cardiovascular risk
ECC: Extracorporeal circulation
ELISA: Enzyme-linked immunosorbent assay
ICU: Intensive care unit
IDDM: Insulin-dependent diabetes mellitus (type II)
DSWI: Deep sternal wound infection
IL-6: Interleukin 6
LVEF: Left ventricular ejection fraction
3-NT: 3-nitrotyrosine
SD (in Results): Standard deviation
SEM (Figures 4-7): Standard error of the mean.
